# Watermelon-induced hyperkalemia in chronic kidney disease patients: perspective from Pakistan

**DOI:** 10.1097/MS9.0000000000002626

**Published:** 2024-09-30

**Authors:** Abdullah, Humza Saeed, Muhammad H. Ahmad

**Affiliations:** a Department of Internal Medicine, Rawalpindi Medical University, Rawalpindi, Punjab, Pakistan; b Tentishev Satkynbai Memorial Asian Medical Institute, Kant, Kyrgyzstan

*Dear Editor*,

Chronic kidney disease (CKD) is characterized by a progressive decline in kidney function (<60 ml/min/1.73 m^2^) leading to electrolyte and fluid imbalance with an incidence in Pakistan estimated between 12.5 and 31.2%^1^. Rising cases of end-stage kidney disease (ESKD) underscore CKD’s global morbidity and mortality burden^2^. A 2015 systematic analysis revealed a CKD prevalence of 387.5 million in lower-middle-income countries^3^. CKD increases the risk of end-stage renal disease (ESRD), necessitating costly therapies like dialysis and transplantation^4^. CKD, often stemming from uncontrolled diabetes and hypertension, warrants priority due to its global epidemic status. In Pakistan, CKD prevalence varies by age, with higher rates in older adults. Female prevalence fluctuates based on four studies from Pakistan, yet CKD affects a significant proportion of both genders^5-8^. In South Asia, CKD affects 1–4 out of every 10 individuals, with Pakistan exhibiting the highest prevalence at 21.2% and India the lowest at 10.2%^9^.

Patients with CKD are prone to hyperkalemia due to reduced potassium excretion capacity, medication usage, and metabolic acidosis prevalence^10^. Despite declining glomerular filtration rates, normal serum potassium levels can be maintained in CKD until GFR falls below 15 ml/min, significantly elevating hyperkalemia risk^11^. Identifying the causes of hyperkalemia, including renal function decline and dietary habits, becomes imperative^12^. To maintain the resting membrane potential (RMP) of excitable cells, extracellular potassium concentration is tightly regulated. Protective mechanisms counter hyperkalemia, including increased renal potassium excretion driven by aldosterone and testosterone-induced enhancement of epithelial sodium channels in the collecting duct. Insulin facilitates potassium transcellular changes post-meals, and a feedforward kaliuretic signal from the stomach to the kidney exists, independent of aldosterone^13^.

Pakistan produces millions of tons of watermelon each year. Due to the low prices of the fruit and high demand for watermelon in long duration summers (April–September), this fruit is consumed highly by the people of Pakistan. In individuals without underlying health conditions, over-eating watermelon is improbable to cause dehydration or cardiac complications. However, a recent case series conducted by Sushmita Prabhu *et al*.^14^ illustrates that excessive intake of watermelon may lead to hyperkalemia in individuals with end-stage renal failure or advanced chronic kidney disease (CKD). Similar results were reported in another case report from Iran, which showed that the patient had decreased his BUN and creatinine levels after eating a large amount of watermelon. This was followed by an increase in the patient’s blood level of antioxidants, which in turn had an impact on kidney function^15^. According to a different source, people with hyperkalemia and those with stage three or higher CKD are advised against eating watermelon^16^.

A sizable watermelon slice (15–7.5 cm) can provide about 5060 mg (126 mmol) of potassium, compared to the WHO-recommended daily potassium intake of 3510 mg (90 mmol)^17^. This is a potentially hazardous amount for individuals with compromised kidney function. Despite its reputation as a low-potassium fruit, a watermelon wedge still contains ~320 mg (8 mmol) of potassium. Similarly, 10 scoops of watermelon contain 137 mg of potassium per serving, while 100 ml of watermelon juice equates to 170 mg^18^. This lesser-known potassium source is often disregarded due to seasonal availability and the quantity needed to elicit noticeable effects compared to more recognized high-potassium foods like potatoes, tomatoes, and bananas. Besides its essential benefits of quenching thirst and antioxidant properties, CKD patients need to be careful while taking such fruits as they are at increased risk of hyperkalemia, which can cause heart problems. For the management of hyperkalemia in ESRD, a case was reported that highlighted the importance of maintaining a food journal for such patients to identify hyperkalemia triggers^19^.

Lifestyle modifications for CKD patients encompass dietary management, physical activity, medication adherence, and mental health support. Key dietary adjustments include limiting sodium to control blood pressure, managing protein intake, and regulating potassium and phosphorus levels. Due to potential imbalances, patients should avoid high-potassium foods (e.g. bananas, oranges) and phosphorus-rich items (e.g. dairy, nuts). Hydration is essential, but overhydration, which may strain the kidneys, should be avoided. Moderate exercise, such as walking or cycling, supports cardiovascular health, weight management, and overall well-being, while strenuous activities that may cause dehydration or blood pressure spikes should be avoided. Adherence to prescribed medications, particularly for blood pressure, blood sugar, and anemia, is crucial. Over-the-counter NSAIDs like ibuprofen should be avoided. The psychological impact of chronic illness necessitates access to mental health professionals and support groups. Practices like meditation, yoga, and deep breathing exercises can be beneficial for managing stress^20^.

Policies for CKD patients should focus on healthcare accessibility, education, nutritional support, and research. Governments must ensure that CKD medications are affordable and accessible, particularly for low-income patients. National screening programs for early CKD detection, especially in high-risk groups (e.g. those with diabetes or hypertension), are essential. Increased funding for CKD research is needed to explore innovative treatments, early detection methods, and potential cures. Subsidized healthy food options should be provided, especially for low-income CKD patients, with clear labeling on sodium, potassium, and phosphorus content to facilitate informed dietary choices^21^.

Enhancing medical practices and minimizing errors are critical in CKD management. Regular continuing medical education should include updates on the latest research and treatment protocols. Interdisciplinary collaboration among nephrologists, dietitians, and mental health professionals is vital for holistic care. Patient-centered care should be emphasized, with personalized treatment plans considering the patient’s CKD stage, comorbidities, and lifestyle factors. Effective communication training is essential to ensure patients understand their diagnosis, treatment options, and lifestyle recommendations. Electronic health records (EHRs) should be utilized to track patient progress, medication adherence, and lab results, reducing errors and ensuring continuity of care. Expanding telemedicine services is also necessary, particularly for patients in rural or underserved areas. Error reduction strategies include implementing standardized checklists and treatment protocols, encouraging peer review systems, and promoting case discussions among healthcare providers to minimize diagnostic errors and improve treatment outcomes^22^.

Thus, in countries like Pakistan, which are high producers of watermelon with long summer durations and high prevalence of CKD, special caution should be taken while handling such patients. Clinicians in renal palliative care should provide tailored dietary guidance to prevent electrolyte imbalances and fluid overload in patients opting for conservative therapy. A multifactorial approach to prevent hyperkalemia recurrence should be adopted, such as substituting watermelon with apple juice. Moreover, it is essential to educate and make patients and their attendants aware of the dietary benefits and hazards of watermelon. In conclusion, future high-quality research is needed to clarify the risk factors and underlying associations pertaining the watermelon-induced hyperkalemia.

## Ethical approval

Ethical approval was not required for this correspondence.

## Consent

Informed consent was not required for this correspondence.

## Source of funding

The authors received no extramural funding for the study.

## Author contribution

All authors contributed to the conceptualization, literature review, drafting, editing, and reviewing of this editorial.

## Conflicts of interest disclosure

The authors declare no potential conflicts of interest concerning the research, authorship, and/or publication of this article.

## Research registration unique identifying number (UIN)

Not applicable.

## Guarantor

Not applicable.

## Data availability statement

Not applicable.

## Provenance and peer review

Not applicable.
